# Supplementary material for: The association between smartphone use and sleep quality, psychological distress, and loneliness among health care students and workers in Saudi Arabia
Source: PLoS One. 2023 Jan 26;18(1):e0280681. doi: 10.1371/journal.pone.0280681 (PMC9879389; doi:10.1371/journal.pone.0280681)
Supplement: S1 File — (PDF) [file pone.0280681.s002.pdf]

The association between smartphone use and sleep quality, psychological distress, and loneliness among health care students and workers in Saudi Arabia

Demographic data

1. What is your gender?
    a. Male
    b. Female
2. What is your age ?

3. What is your specialty?
    a. Dentistry
    b. Public health
    c. Pharmacy
    d. Medicine
    e. Nursing
4. What is your qualification?
    a. Student
    b. Intern
    c. Graduate (bachelor
    d. Specialist
    e. Consultant
5. What is your City?

6. What is your region in Saudi Arabia?
    a. Western
    b. Central
    c. Southern
    d. Eastern
    e. Northern
7. What is your nationality?

a. Saudi
b. Non-Saudi

8. Please answer the following question about general health:
    a. Do you have any chronic disease? (yes, no)
    b. Do you walk regularly? (yes, no)
    c. Do you eat health food regularly? (yes, no)

9. Please choose the appropriate choice in relation to your smartphone use habits. 1 (strongly disagree), 2, 3, 4, 5(strongly Agree).
    a. My smartphone is the most important thing in my life.
    b. Conflicts have arisen between me and my family (or friends) because of my smartphone use.
    c. Preoccupying myself with my smartphone is a way of changing my mood (I get a buzz, or I can escape or get away, if I need to).
    d. Over time, I fiddle around more and more with my smartphone.
    e. If I cannot use or access my smartphone when I feel like, I feel sad, moody, or irritable.
    f. If I try to cut the time I use my smartphone, I manage to do so for a while, but then I end up using it as much or more than before.

10. Sleep Quality assessment: During the past 7 days, how would you rate your sleep quality overall? (Such as how many hours of sleep you got, how easily you fell asleep, how often you woke up during the nights (except to go to the bathroom), how often you woke up earlier than you had to in the morning, and how refreshing your sleep was).

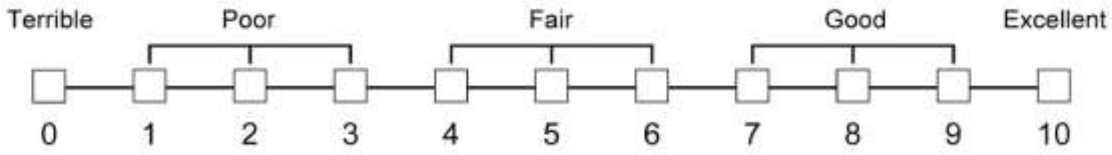

11. Please choose the correct choice: In the past 4 weeks, about how often did you feel (None of the time (1), A little of the time (2), Some of the time (3), Most of the time (4), All of the time (5))
    a. tired out for no good reason?
    b. nervous?
    c. so nervous that nothing could calm you down?
    d. hopeless?
    e. restless or fidgety?
    f. so restless you could not sit still?
    g. depressed?
    h. that everything was an effort?
    i. so sad that nothing could cheer you up?
    j. worthless?

12. Loneliness: Please answer the following question.(No, More or less, Yes)
    a. I experience a general sense of emptiness.
    b. There are plenty of people I can rely on when I have problems.
    c. There are many people I can trust completely.
    d. I miss having people around.
    e. There are enough people I feel close to.
    f. I often feel rejected.
